# Clinical implications of circulating follistatin-like protein-1 in hemodialysis patients

**DOI:** 10.1038/s41598-023-33545-w

**Published:** 2023-04-24

**Authors:** Dae Kyu Kim, Seok Hui Kang, Jin Sug Kim, Yang Gyun Kim, Yu Ho Lee, Dong-Young Lee, Shin Young Ahn, Ju Young Moon, Sang Ho Lee, Kyung Hwan Jeong, Hyeon Seok Hwang

**Affiliations:** 1 Division of Nephrology, Department of Internal Medicine, College of Medicine, Kyung Hee University Hospital, Kyung Hee University, Seoul, Republic of Korea; 2 Division of Nephrology, Department of Internal Medicine, College of Medicine, Yeungnam University Medical Center, Daegu, Republic of Korea; 3 Division of Nephrology, Department of Internal Medicine, College of Medicine, Kyung Hee University Hospital at Gangdong, Seoul, Republic of Korea; 4 Division of Nephrology, Department of Internal Medicine, CHA Bundang Medical Center, CHA University, Seongnam, Republic of Korea; 5 Division of Nephrology, Department of Internal Medicine, Veterans Health Service Medical Center, Seoul, Republic of Korea; 6 Division of Nephrology, Department of Internal Medicine, College of Medicine, Korea University, Seoul, Republic of Korea

**Keywords:** Renal replacement therapy, Cardiovascular biology, Predictive markers

## Abstract

Follistatin-like protein-1 (FSTL-1) is secreted glycoprotein, which regulates cardiovascular, immune and skeletal system. However, the clinical significance of circulating FSTL-1 levels remains unclear in hemodialysis patients. A total 376 hemodialysis patients were enrolled from June 2016 to March 2020. Plasma FSTL-1 level, inflammatory biomarkers, physical performance, and echocardiographic findings at baseline were examined. Plasma FSTL-1 levels were positively correlated with TNF-α and MCP-1. Handgrip strength showed weak positive correlation in male patients only, and gait speed showed no correlation with FSTL-1 levels. In multivariate linear regression analysis, FSTL-1 level was negatively associated with left ventricular ejection fraction (*β* =  − 0.36; *p* = 0.011). The cumulative event rate of the composite of CV event and death, and cumulative event rate of CV events was significantly greater in FSTL-1 tertile 3. In multivariate Cox-regression analysis, FSTL-1 tertile 3 was associated with a 1.80-fold risk for the composite of CV events and death(95% confidence interval (CI) 1.06–3.08), and a 2.28-fold risk for CV events (95% CI 1.15–4.51) after adjustment for multiple variables. In conclusion, high circulating FSTL-1 levels independently predict the composite of CV events and death, and FSTL-1 level was independently associated with left ventricular systolic dysfunction.

## Introduction

Follistatin-like protein-1 (FSTL-1) is an extracellular glycoprotein that is widely expressed in the human cardiovascular (CV), immune, and musculoskeletal systems^1^. FSTL1 is weakly expressed in the normal myocardium and quiescent inflammatory cells, although it is strongly induced in their pathologic conditions, including hypertrophic cardiomyocytes and activated inflammatory cells^2,3^. Additionally, these organs and systems secrete FSTL-1, which exists in a soluble form in the bloodstream. Circulating FSTL-1 levels are increased in patients with atherosclerotic CV disease, ischemic cardiomyopathy, and several autoimmune diseases^4–7^. Physical exercise training also increases the skeletal expression of FSTL-1 concomitantly with higher circulating FSTL-1 levels^8–11^.

Hemodialysis (HD) patients have an extremely high risk of CV complications^12,13^. Left ventricular (LV) hypertrophy, cardiac ischemia, and a higher status of inflammation are commonly observed in HD patients, and these pathological factors increase the risk of atherosclerotic events in such patients^14,15^. Additionally, substantial retention of uremic toxins and inflammatory molecules causes constant loss of skeletal muscle^16^. Over 70% of HD patients have lower muscle mass and strength^17–19^. These pathologic conditions in HD patients contribute not only to worse physical health but also to adverse clinical outcomes, including CV complications and death^20,21^. Considering the multiple functions of FSTL-1, such as a cardiokine, myokine, and inflammatory modulator, circulating FSTL-1 may be a promising biomarker for determining the status of CV, inflammation, and physical performance and predicting adverse clinical outcomes in HD patients.

However, the clinical significance of FSTL-1 in HD patients has rarely been reported, and no studies have investigated the potential role of FSTL-1 as a CV biomarker and an indicator of physical performance. In this study, we investigated the association between circulating FSTL-1 levels and the risk of incident CV events, and evaluated physical performance to determine its relationship with FSTL-1 levels.

## Methods

### Study population

The data used this study were obtained from the K-cohort registry. The K-cohort is a multicenter, prospective cohort of HD patients in Korea designed to investigate prognostic markers of CV complications and death^15,22,23^. Patients aged > 18 years who received regular 4-h HD prescriptions per session thrice a week for at least 3 months were included in the study. The exclusion criteria were pregnancy, hematological malignancy, presence of a solid tumor, and a life expectancy of < 6 months. After applying the exclusion critieria, 376 patients with plasma samples collected at the time of study enrollment from June 2016 to March 2020 in five hospitals were included in the study. There were no patients with acute infectious condition at the time of enrollment. The patients were classified into three groups based on plasma FSTL-1 levels as follows: tertile 1, < 18.00 ng/ml; tertile 2, 18.00–20.14 ng/ml; and tertile 3 ≥ 20.14 ng/ml. Patient follow-up was censored at the time of transfer to peritoneal dialysis, kidney transplantation, follow-up loss, or patient consent withdrawal.

### Ethics approval and consent to participate

The study protocol was approved by the Institutional Review Board of Kyung Hee University Medical Center (IRB number: 2016-04-039), and the study was conducted in accordance with the principles of the second Declaration of Helsinki. All study participants provided written informed consent before enrollment.

### Data collection and definitions

Information on baseline demographic factors, laboratory results, dialysis, and concomitant medications was collected through patient interviews and a review of medical records at the time of inclusion. BMI was defined as body weight divided by the square of body height. Charlson comorbidity index scores were calculated, and blood samples for laboratory tests and other markers were collected before the start of HD in a mid-week dialysis session^24^. The delivered spKt/V (K, dialyzer clearance; t, time; V, urea distribution volume) was assessed using the conventional method.

### Outcome measures

The primary endpoint of the study was the composite of incident CV events and death. Cardiac events were defined as coronary artery disease requiring coronary artery bypass surgery or percutaneous intervention, myocardial infarction, ventricular arrhythmia, cardiac arrest, or sudden death. Non-cardiac events included cerebral infarction, cerebral hemorrhage, and peripheral vascular occlusive diseases that required revascularization or surgical intervention. All-cause deaths were recorded. Secondary endpoints were inflammatory biomarkers, physical performance, and echocardiographic parameters, which were correlated with FSTL-1 level.

### Echocardiographic measurements

Of all the patients, 337 (89.6%) underwent echocardiography [110 (87.3%) in tertile 1, 114 (91.2%) in tertile 2, and 113 (90.4%) in tertile 3]. Two-dimensional and M-mode echocardiography was performed by cardiologists and trained sonographers based on the recommendations of the American Society of Echocardiography^25^. LV end-diastolic diameter, LV end-systolic diameter (LVDs), LV posterior wall thickness, and interventricular septal thickness were measured by M-mode echocardiography. LV mass was estimated using the Devereux formula with body surface area as the index. LV end-diastolic and LV end-systolic volumes, LV ejection fraction (LVEF), and left atrial dimensions were determined in apical two- and four-chamber views. Peak early (E) and late diastolic mitral inflow velocity (A) was determined from the mitral valve inflow velocity curve by pulsed-wave Doppler ultrasonography. Early diastolic mitral annular tissue velocity (E′) was measured from the septal aspect of the mitral annulus using tissue Doppler imaging.

### Laboratory measurements

Plasma samples for the measurement of FSTL-1 levels were collected in ethylenediaminetetraacetic acid-treated tubes at study inclusion. After centrifugation at 1000 g for 15 min at room temperature, the samples were stored at − 80 °C until use. An enzyme-linked immunosorbent assay was performed using Magnetic Luminex® Screening Assay multiplex kits (R&D Systems Inc., Minneapolis, MN, USA) to measure FSTL-1, tumor necrosis factor-alpha (TNF-α), and monocyte chemoattractant protein-1 (MCP-1). TNF-α and MCP-1 levels were measured in 280 (74.5%) patients owing to sample availability.

### Physical performance measurements

Handgrip strength and gait speed were measured to investigate physical performance based on the suggestions of the Asian Working Group for Sarcopenia^26^. Handgrip strength was measured using a Jamar hand dynamometer (Sammons Preston, Bolingbrook, IL) on the dominant hand during dialysis sessions. Each measurement was repeated thrice, and the highest value was recorded^27^. Gait speed was assessed by measuring the walking speed over a 4-m course at the patient’s usual pace. The measurement was repeated thrice, and the average speed was recorded. Physical performance was examined in 296 (78.7%) patients.

### Statistical analysis

Data are expressed as mean ± standard deviation or median (interquartile range [IQR]). Differences between more than three groups were identified using analysis of variance or the Kruskal–Wallis test. Tukey’s post hoc test and the Mann–Whitney *U*-test were used to identify differences between more than two groups. Categorical variables were compared using the chi-squared test or Fisher’s exact test. High-sensitivity C-reactive protein (hsCRP) levels were log-transformed in the regression analysis because of a skewed distribution. Spearman’s correlation test was used to evaluate the correlation between FSTL-1 and continuous variables. The association between FSTL-1 levels and LV systolic and diastolic function was evaluated using linear regression analysis. Cumulative event rates were estimated using the Kaplan–Meier method and compared using the log-rank test. A Cox proportional hazards model was constructed to identify the relationship between FSTL-1 level and the risk of composite CV events and death. Significantly associated parameters in the univariate analysis and clinically meaningful parameters were included in the multivariate analysis. Adjusted variables in the multivariate linear regression analysis were age, sex, BMI, history of CV events, diabetes, hsCRP levels, and dialysis duration. Age; sex; BMI; Charlson comorbidity index; spKt/V; and levels of hemoglobin, hsCRP, low-density lipoprotein cholesterol and intact parathyroid hormone were included in the multivariate Cox regression test. Statistical analyses were performed using the SPSS software (version 22.0; SPSS, IBM Corp., Armonk, NY, USA). Statistical significance was set at *p* < 0.05.

## Results

### Baseline demographic characteristics and laboratory data

The median FSTL-1 level was 19.01 (IQR 17.13, 20.90) ng/ml. Based on tertiles, the median FSTL-1 level was 16.56 (IQR 15.25, 17.15) ng/ml in tertile 1, 19.01 (IQR 18.45, 19.57) ng/ml in tertile 2, and 22.08 (IQR 20.90, 23.64) ng/ml in tertile 3. Table 1 presents the baseline characteristics of the study population. Patient demographics, comorbidities, laboratory findings, and dialysis characteristics did not differ across the tertiles. Among the inflammatory markers, patients in tertile 3 had higher MCP-1 and TNF-α levels than those in tertile 1 and tertile 2, respectively.

**Table 1:** Baseline demographic and laboratory data of the study population.

	Tertiles of circulating FSTL-1 (ng/ml)			*p* value
	Tertile 1 < 18.0 ng/ml (n = 126)	Tertile 2 18.0–20.1 ng/ml (n = 125)	Tertile 3 > 20.1 ng/ml (n = 125)	
Age (years)	62.0 ± 12.4	60.8 ± 13.2	60.0 ± 12.3	0.442
Male (%)	81 (64.3)	84 (67.2)	80 (64.0)	0.841
History of CV event (%)	47 (37.3)	53 (42.4)	56 (44.8)	0.475
HD duration (year)	3.9 ± 5.2	4.0 ± 5.6	3.6 ± 5.7	0.845
Charlson comorbidity index	4.0 ± 1.5	4.0 ± 1.5	4.2 ± 1.5	0.475
Diabetes (%)	72 (57.1)	65 (52.0)	74 (59.2)	0.497
Body mass index (kg/m^2^)	23.9 ± 3.8	23.0 ± 4.3	23.1 ± 4.3	0.149
Albumin (g/l)	3.8 ± 0.3	3.8 ± 0.3	3.8 ± 0.3	0.171
TIBC (mg/dl)	219.5 ± 39.1	224.5 ± 36.6	225.2 ± 39.4	0.445
Hemoglobin (g/dl)	10.5 ± 1.3	10.6 ± 1.1	10.4 ± 1.3	0.598
Ca (mg/dl)	8.5 ± 0.8	8.5 ± 0.8	8.4 ± 0.8	0.256
Phosphorus (mg/dl)	4.9 ± 1.2	4.9 ± 1.3	5.1 ± 1.4	0.494
i-PTH (pg/ml)	239.7 ± 172.3	322.0 ± 251.0^a^	319.0 ± 230.7^a^	0.004
LDL-cholesterol (mg/dl)	78.2 ± 26.7	77.1 ± 27.3	74.2 ± 24.4	0.460
Pre-dialysis SBP (mmHg)	142.7 ± 19.3	140.7 ± 18.9	144.8 ± 19.9	0.257
spKt/V	1.6 ± 0.3	1.6 ± 0.3	1.6 ± 0.3	0.866
TNF-α (pg/ml)	10.2 (6.8, 13.2)	10.4 (6.9, 13.2)	11.3 (8.6, 14.8)^b^	0.016
hsCRP (mg/dl)	0.9 (0.2, 3.6)	1.2 (0.3, 3.3)	1.4 (0.4, 4.3)	0.563
MCP-1 (pg/ml)	153 (125, 195)	170 (131, 224)	169 (145, 241)^a^	0.007

Data are expressed as mean ± SD or median (interquartile range).

TNF-α and MCP-1 were measured in 280 (74.5%) patients.

*FSTL-1* follistatin-like protein-1, *CV* cardiovascular, *HD* hemodialysis, *Ca* calcium, *TIBC* total iron binding capacity, *i-PTH* intact parathyroid hormone, *LDL-cholesterol* low-density lipoprotein cholesterol, *SBP* systolic blood pressure, *spKt/V* single-pool Kt/V, *TNF-α* tumor necrosis factor-α, *hsCRP* high-sensitivity C-reactive protein, *MCP-1* monocyte chemoattractant protein-1.

^a^*p* < 0.05 vs. textile 1.

^b^*p* < 0.05 vs. tertile 2.

### Correlation of FSTL-1 level with physical performance and inflammatory markers

The baseline physical performance of the study population is described in Additional file 1 (Table S1). Male patients in FSTL-1 tertile 3 had higher handgrip strength than those in FSTL-1 tertile 1. However, the gait speed did not differ across tertiles. Table 2 presents the correlation between the FSTL-1 and physical performance. Handgrip strength was weakly correlated in the male patients only (*β* = 0.148; *p* = 0.038), and gait speed demonstrated no correlation with FSTL-1 levels.

**Table 2:** Correlation of circulating FSTL-1 level with physical performance.

Physical performance	Correlation coefficient	*p* value
Gait speed (m/s)		
Male	− 0.016	0.842
Female	− 0.028	0.803
Handgrip strength (kg)		
Male	0.148	0.038
Female	− 0.020	0.845

Gait speed and hand grip were examined in 294 (78.2%) patients.

*FSTL-1* follistatin-like protein-1.

The correlation between FSTL-1 and inflammatory markers was evaluated. TNF-α (*β* = 0.125; *p* = 0.036) and MCP-1 (*β* = 0.185; *p* = 0.002) levels had weak positive correlation with FSTL-1 levels.

### Relationship between plasma FSTL-1 levels and echocardiographic parameters

Baseline echocardiographic measurements are described in Additional file 2 (Table S2). Patients in FSTL-1 tertile 3 had significantly longer LVDs, higher LV end-systolic volume, and lower LVEF than those in tertile 1. Univariate and multivariate linear regression models were constructed to determine the association of FSTL-1 levels with LV systolic and diastolic functions (Table 3). In the univariate analysis, FSTL-1 per standard deviation (SD) and FSTL-1 per tertile were significantly associated with LVEF. E/E′ also demonstrated a significant association with FSTL-1 per SD. In the multivariate analysis, LVEF was independently associated with FSTL-1 per SD (*β* =  − 1.13; *p* = 0.011) and FSTL-1 per tertile (*β* =  − 1.67; *p* = 0.002). However, FSTL-1 levels were not significantly associated with E/E′.

**Table 3:** Relationship between FSTL-1 and left ventricle systolic and diastolic functions.

	Univariate analysis		Multivariate analysis	
	Unstandardized *β *(95% CI)	*p* value	Unstandardized *β *(95% CI)	*p* value
LVEF				
FSTL-1 per SD	− 1.23 (− 2.09, − 0.37)	0.005	− 1.13 (− 2.00, − 0.26)	0.011
FSTL-1 per tertile	− 1.82 (− 2.88, − 0.77)	0.001	− 1.67 (− 2.73, − 0.60)	0.002
E/E´				
FSTL-1 per SD	0.84 (0.10, 1.56)	0.025	0.61 (− 0.11, 1.33)	0.096
FSTL-1 per tertile	0.86 (− 0.06, 1.77)	0.066	0.48 (− 0.41, 1.38)	0.287

All analyses were adjusted for the following covariates: age, sex, BMI, history of CV events, diabetes, hsCRP level, and dialysis duration.

*FSTL-1* follistatin-like protein-1, *BMI* body mass index, *CV* cardiovascular, *hsCRP* high-sensitivity C-reactive protein, *E/E´* early diastolic mitral inflow velocity/late diastolic mitral inflow velocity, *LVEF* left ventricular ejection fraction, *SD* standard deviation.

### Prognostic utility of FSTL-1 levels for CV events

**Figure 1:**
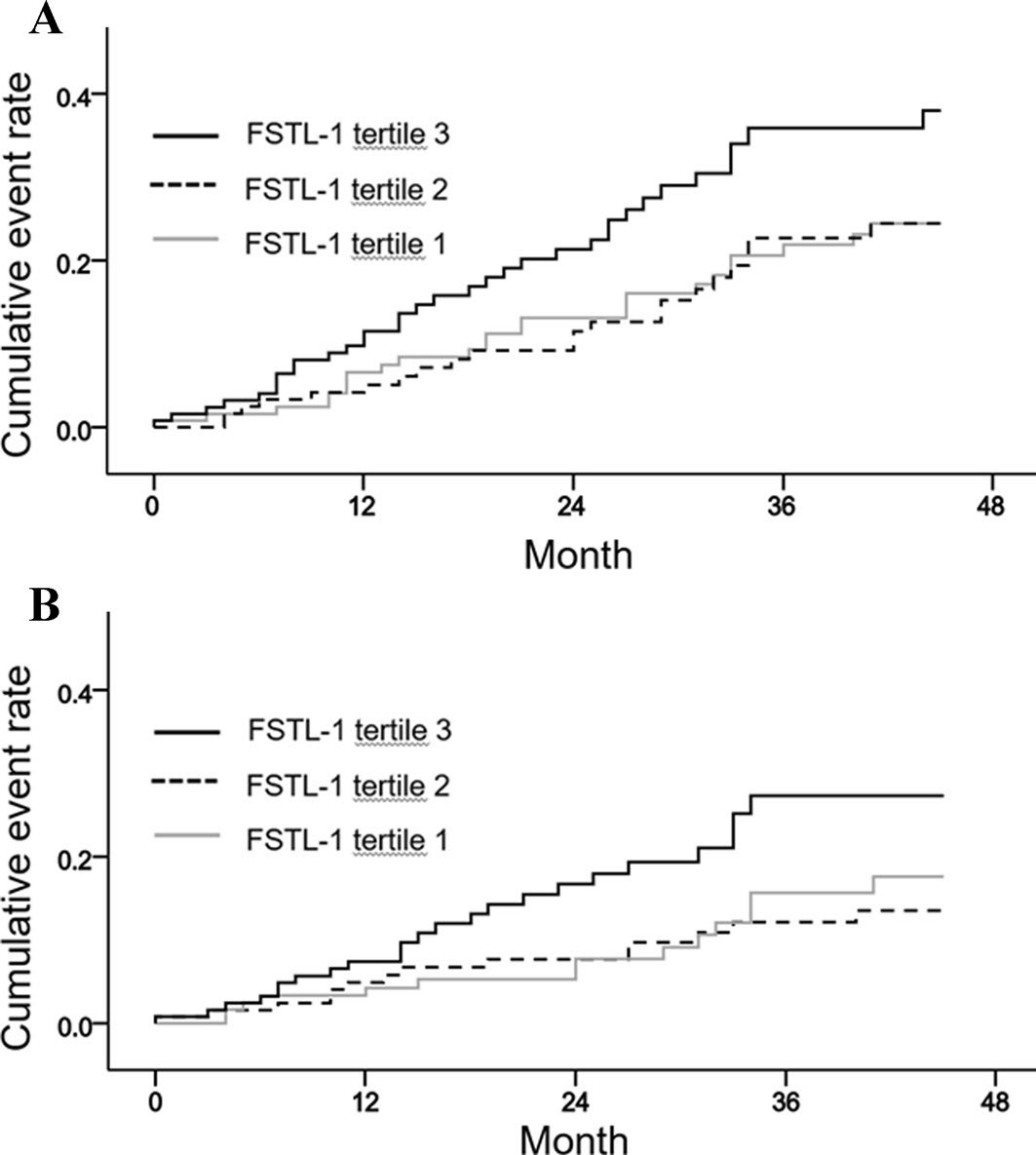
Cumulative event rate of the composite of cardiovascular events and death (**A**) and cardiovascular events (**B**) according to follistatin-like protein-1 (FSTL-1) levels.

In total, 52 (13.8%) deaths and 51 (13.6%) CV events occurred during a mean follow-up of 30.4 ± 14.3 months, and 37 patients were lost to follow-up. Coronary artery disease occurred in 19 patients, ventricular arrhythmia in 3, cardiac arrest in 7, sudden death in 5, cerebral vascular accidents in 10, and peripheral vascular occlusive diseases in 7. Patients in FSTL-1 tertile 3 had the highest cumulative rate of composite CV events and death (*p* = 0.037; Fig. 1A) and a greater cumulative rate of CV events (*p* = 0.048; Fig. 1B). Patient mortality rate did not differ between the FSTL-1 tertiles (*p* = 0.747).

The univariate Cox regression analysis revealed that plasma FSTL-1 tertile 3 was significantly associated with an increased risk of a composite of CV events and death [hazard ratio (HR) 1.74; 95% confidence interval (CI) 1.04–2.93; *p* = 0.036; Table 4]. This association remained significant after adjusting for multiple variables (HR 1.80; 95% CI 1.06–3.08; *p* = 0.029). FSTL-1 increment per 1 SD was independently associated with an increased risk of a composite of CV events and death (HR 1.30; 95% CI 1.04–1.62; *p* = 0.021). To further investigate the risk of CV events, HRs for CV events were evaluated. In the multivariable Cox regression analysis, the patients in FSTL-1 tertile 3 had a 2.28-fold risk of CV events (1.15–4.51; *p* = 0.018), and the FSTL-1 increment per 1 SD was also associated with a higher risk of CV events (HR 1.39; 95% CI 1.05–1.83, *p* = 0.019).

**Table 4:** Hazard ratios for a composite of CV events and death, CV events, and all-cause deaths.

	No. of events (%)	Univariate analysis	Multivariate analysis
		HR (95% CI)	HR (95% CI)
Composite of CV events and death			
FSTL-1 tertile 1	25 (19.8)	Reference	
FSTL-1 tertile 2	21 (16.8)	0.97 (0.54–1.73)	1.07 (0.59–1.96)
FSTL-1 tertile 3	34 (27.2)	1.74 (1.04–2.93)	1.80 (1.06–3.08)
FSTL-1 per SD		1.24 (1.01–1.53)	1.30 (1.04–1.62)
CV events			
FSTL-1 tertile 1	14 (11.1)	Reference	
FSTL-1 tertile 2	14 (11.2)	1.15 (0.55–2.42)	1.39 (0.65–2.99)
FSTL-1 tertile 3	23 (18.4)	2.10 (1.08–4.10)	2.28 (1.15–4.51)
FSTL-1 per SD		1.29 (1.00–1.67)	1.39 (1.05–1.83)
Patient deaths			
FSTL-1 tertile 1	18 (14.3)	Reference	
FSTL-1 tertile 2	14 (11.2)	0.90 (0.45–1.80)	0.88 (0.42–1.82)
FSTL-1 tertile 3	20 (16.0)	1.38 (0.73–2.62)	1.47 (0.76–2.84)
FSTL-1 per SD		1.16 (0.89–1.52)	1.20 (0.90–1.59)

All analyses were adjusted for the following covariates: age; sex; BMI; Charlson comorbidity index; spKt/V; and levels of hemoglobin, hsCRP, LDL cholesterol and i-PTH.

*FSTL-1* follistatin-like protein-1, *BMI* body mass index, *hsCRP* high-sensitivity C-reactive protein, *LDL-cholesterol* low-density lipoprotein cholesterol, *i-PTH* intact parathyroid hormone, *no.* number, *HR* hazard ratio, *CI* confidence interval, *SD* standard deviation.

## Discussion

Our study used a prospective cohort dataset of incident and prevalent HD patients. Our results revealed a positive association between FSTL-1 levels and two inflammatory indicators, TNF-α and MCP-1 and a negative association with hsCRP levels. Patients with high FSTL-1 levels had higher LVDs, LV end-systolic volumes, and lower LVEF than those with low FSTL-1 levels. Furthermore, an increase in FSTL-1 SD or tertile was associated with a composite of CV events and death. We also evaluated the association of FSTL-1 levels with gait speed and handgrip strength. Handgrip strength was weakly associated with FSTL-1 levels in male patients.

FSTL-1 is an extracellular glycoprotein that is secreted in various tissues, such as cardiomyocytes, osteoblastic cells, and skeletal muscles^8,28,29^. Prior studies have found that serum FSTL-1 levels were elevated in patients with CV diseases, including heart failure and acute coronary syndrome^7,29,30^. High FSTL-1 levels in patients with these pathologies are associated with patient mortality. Thus, FSTL-1 could be a potential new prognostic biomarker for patients with CV disease or damage. Additionally, recent studies have demonstrated the protective or regenerative capacities of FSTL-1 levels in damaged tissues rather than the hazardous effect of FSTL-1, and their potential as a new therapeutic option has been evaluated^31^. Our study included HD patients who were exposed to various pathologic conditions, such as uremic toxins, volume overload, or anemia^32^. HD patients had a higher prevalence of CV disease than the general population. Therefore, the identification of new indicators for CV disease or death is important for improving the prognosis of HD patients. HD patients are frequently exposed to myocardial ischemia and damage during or after HD, and this silent myocardial damage may be associated with increased FSLT-1 levels. In our study, patients with high FSTL-1 levels had greater LVDs, LV end-systolic volumes, and poorer LVEF than those with low FSTL-1 levels. Consequently, this would lead to the development of a composite of CV events and death. Our study suggests FSTL-1 levels as a novel indicator for predicting CV outcomes in HD patients.

We observed that a higher FSTL-1 level was independently associated with a lower LVEF, and that a higher tertile of FSTL-1 level was significantly associated with the increase in LVDs and LVESV, but not with LVDd and LVEDV. These findings suggest that FSTL-1 is a reliable indicator of impaired LV contraction, and that FSTL-1 levels are more closely related to pathological systolic deformation than to diastolic deformation. Notably, we found no relationship between FSTL-1 level and E/E’ ratio in multivariate analysis, and the E/E’ ratio did not differ across FSTL-1 tertiles. These findings further support the notion that circulating FSTL-1 levels are more reflective of systolic dysfunction than diastolic dysfunction in HD patients. Therefore, we suggest that circulating FSTL-1 is more appropriate as a marker of cardiac systolic dysfunction.

In our study, we observed a positive association between TNF-α or MCP-1 and FSTL-1, but a negative association between hsCRP and FSTL-1, despite the fact that all of them are widely recognized as inflammatory biomarkers. This discrepancy may be due to differences in the characteristics of these factors. TNF-α and MCP-1 are cytokines/chemokines that are directly excreted through inflammatory cells and have short half-lives (18.2 min for TNF-α and < 20 min for MCP-1)^33–35^, while hsCRP is an acute phase protein synthesized in the liver in response to inflammation and has a longer half-life of 19–20 h^36^. Therefore, TNF-α and MCP-1 may be less influenced by events during the inter-dialysis period than hsCRP, which could explain the decreased association between hsCRP and FSTL-1. However, we recognize the need for further investigation using repeated measurements and larger sample sizes to establish a clear association among these variables. We have added this explanation to the discussion section.

Skeletal muscles are the main source of FSLT-1, and FSTL-1 promotes endothelial cell function and revascularization in ischemic muscles^37^. HD is highly prevalent in patients with low strength and/or low muscle mass. Therefore, we hypothesized that FSTL-1 has a favorable effect on the regeneration of injured muscle mass or improvement in muscle quality in HD patients. However, gait speed in both sexes and handgrip strength in female patients were not significantly associated with FSTL-1 levels, and a weak positive correlation between FSTL-1 and handgrip strength was observed in male patients. These findings suggest that the relationship between circulating FSTL-1 and muscle power is not strong in HD patients. Physical performance or strength in HD patients can be influenced by many factors, such as aging, malnutrition, myostatin, uremic toxins, volume status, and inflammation^16^. Considering these factors, multifactorial interventions are recommended to improve muscle strength or function in HD patients. Therefore, observing an association between FSTL-1 and muscle strength or physical performance would be difficult without controlling for these confounders. As studies on the association between FSTL-1 and physical performance are few, our results are preliminary for further investigation of the definite association between the two variables.

This study has several limitations. First, our study had a relatively short median follow-up duration of 30.4 months. Second, data on TNF-α, MCP-1, physical performance, and echocardiography were not obtained for all the studied patients, which may have led to bias. Third, we did not reflect the effect of dialysis modalities on FSTL-1 levels, because dialyzability and elimination kinetics of FSTL-1 is not established for different types of dialysis or membranes. While we collected blood samples for FSTL-1 before the start of HD during a mid-week dialysis session to minimize the effect of dialysis modalities or membrane, potential bias may be present. Fourth, our study obtained FSTL-1 levels through a single measurement, it may not fully capture the true level of FSTL-1.

In conclusion, the present study showed that plasma level of FSTL-1 had weak relationship with inflammatory biomarkers and physical performance. FSTL-1 levels were independently associated with LV systolic dysfunction and higher circulating FSTL-1 levels predicted the composite of incident CV events and death in HD patients. These findings reveal that monitoring of FSTL-1 is valuable to assess the risk of CV complication in HD patients and that HD patients with high FSLT-1 are target population for improving prognosis.

## Supplementary Information

Supplementary Tables.

## Data Availability

The datasets generated during and/or analyzed during the current study are available from the corresponding author on reasonable request.
