# Supplementary material for: Clinical implications of circulating follistatin-like protein-1 in hemodialysis patients
Source: Sci Rep. 2023 Apr 24;13:6637. doi: 10.1038/s41598-023-33545-w (PMC10126138; doi:10.1038/s41598-023-33545-w)
Supplement: Supplementary file 1 — Supplementary Tables. [file 41598_2023_33545_MOESM1_ESM.docx]

**Supplementary Tables**

**Table S1.** Baseline mental and physical performance data of the study population.

|  | Tertiles of circulating FSTL-1 | | |  |
| --- | --- | --- | --- | --- |
|  | Tertile 1  <18.0 ng/ml  (n = 106) | Tertile 2  18.0–20.1 ng/ml  (n = 93) | Tertile 3  >20.1 ng/ml  (n = 95) | *p* value |
| Physical performance |  | | | |
| Gait speed (m/s) | 1.0  (0.8, 1.2) | 1.0  (0.8, 1.2) | 1.0  (0.8, 1.2) | 0.945 |
| Male | 1.0  (0.8, 1.2) | 1.1  (0.8, 1.2) | 1.0  (0.8, 1.2) | 0.625 |
| Female | 1.0  (0.9 1.2) | 1.0  (0.8, 1.1) | 1.0  (0.8, 1.2) | 0.773 |
| Handgrip strength | 19.0  (14.0, 27.0) | 19.0  (14.5, 28.5) | 24.0  (16.0, 31.0) | 0.063 |
| Male | 21.0  (14.0, 29.0) | 24.0  (15.0, 34.0) | 28.0^a^  (21.0, 33.0) | 0.046 |
| Female | 16.0  (12.3, 21.0) | 17.0  (14.0, 19.3) | 17.5  (11.3, 23.8) | 0.949 |

Data are expressed as median (interquartile range).

Gait speed and handgrip strength were examined in 294 patients (78.2%).

FSTL-1, follistatin-like protein-1.

^a^*p* < 0.05 vs. tertile 1.

**Table S2.** Baseline echocardiographic parameters of the study population.

|  | Tertiles of circulating FSTL-1 | | |  |
| --- | --- | --- | --- | --- |
|  | Tertile 1  <18.0 ng/ml  (n = 110) | Tertile 2  18.0–20.1 ng/ml  (n = 114) | Tertile 3  >20.1 ng/ml  (n = 113) | *p* value |
| LV mass index (g/m^2^) | 114.7±27.8 | 120.4±39.0 | 122.6±38.7 | 0.386 |
| LVDs (mm) | 32.3±5.6 | 33.0±5.9 | 35.2±9.3 | 0.016 |
| LVDd (mm) | 50.6±5.6 | 51.2±6.7 | 51.9±6.9 | 0.376 |
| LVESV (ml) | 35.0±14.5 | 36.8±16.0 | 42.6±25.0 | 0.037 |
| LVEDV (ml) | 92.1±29.0 | 94.8±29.9 | 102.9±.39.5 | 0.110 |
| LVEF (%) | 64.2±7.4 | 62.9±6.8 | 60.5±9.6 | 0.003 |
| IVST (mm) | 10.1±2.0 | 10.2±1.8 | 10.2±1.7 | 0.915 |
| PWT (mm) | 10.4±1.8 | 10.2±3.5 | 10.1±1.7 | 0.677 |
| E/E´ | 12.6±5.4 | 13.1±5.7 | 14.3±4.9 | 0.164 |
| E/A | 0.8±0.2 | 0.9±0.3 | 1.1±1.4 | 0.072 |

Data are expressed as mean ± standard deviation.

Echocardiography was performed in 337 (89.6%) patients.

E/A, early diastolic mitral inflow velocity/late diastolic mitral inflow velocity; E/E´, early diastolic mitral inflow velocity/early diastolic mitral annular tissue velocity; FSTL-1, follistatin-like protein-1; LA, left atrium; LV, left ventricle; LVDd, left ventricular end-diastolic diameter; LVDs, left ventricular end-systolic diameter; LVEDV, left ventricular end-diastolic volume; LVEF, left ventricular ejection fraction; LVESV, left ventricular end-systolic volume; IVST, interventricular septal thickness in diastole; PWT, posterior wall thickness;
